# Long non-coding RNA HEIH: a novel tumor activator in multiple cancers

**DOI:** 10.1186/s12935-021-02272-5

**Published:** 2021-10-24

**Authors:** Jie-yu Sun, Ming-ming Ni

**Affiliations:** 1 Department of Pharmacy, The First Affiliated Hospital of Nanjing Medical University, Nanjing, 210029 People’s Republic of China; 2 Department of Pharmacy, Children’s Hospital of Nanjing Medical University, 72 Guangzhou Rd., Nanjing, 210008 People’s Republic of China

**Keywords:** HEIH, lncRNA, Cancers, Therapeutic target

## Abstract

The last decade has witnessed the altered expression levels of long non-coding RNA HEIH in different types of cancer. More than half of the HEIH studies in cancer have been published within the last two years. To our knowledge, this is the first review to discuss very recent developments and insights into HEIH contribution to carcinogenesis. The functional role, molecular mechanism, and clinical significance of HEIH in human cancers are described in detail. The expression of HEIH is elevated in a broad spectrum of cancers, and its disorder contributes to cell proliferation, migration, invasion, and drug resistance of cancer cells through different underlying mechanisms. In addition, the high expression of HEIH is significantly associated with advanced tumor stage, tumor size and decreased overall survival, suggesting HEIH may function as a prognostic biomarker and potential therapeutic target for human cancers.

## Introduction

**Fig. 1:**
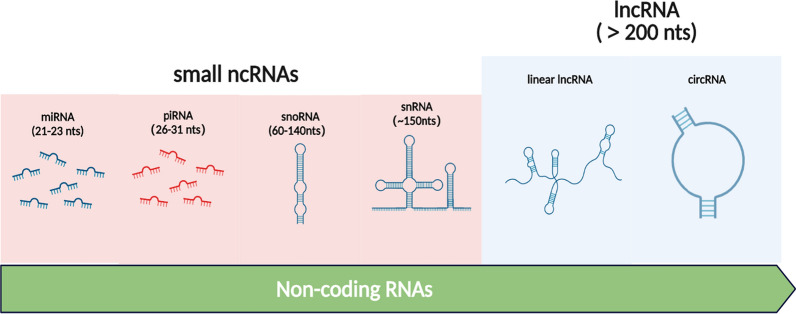
Schematic representation of ncRNAs of different lengths. Small ncRNAs are shorter than 200 nucleotides, including miRNAs (21–23 nucleotides), piRNAs (26–31 nucleotides), snoRNAs (60–140 nucleotides), and snRNAs (~ 150 nucleotides). In contrast to small ncRNAs, lncRNAs are longer than 200 nucleotides, reaching up to thousands of nucleotides of length. CircRNAs are a peculiar group of endogenous lncRNAs molecules, consisting of at least a few hundred nucleotides

Cancer remains one of the major leading causes of mortality and morbidity worldwide due to delayed diagnosis, poor prognosis, high rates of recurrence and continuously emerging drug resistance [1]. Currently, the tools available for diagnosis, prognosis and therapy of cancer is suboptimal, improved strategies for therapeutic interventions including the identification of novel diagnostic and prognostic biomarkers are urgent [2]. Traditionally, somatic mutations in protein-coding genes were thought as the driving force of cancer development and occurrence. Nevertheless, over the past decade, some gaps in the knowledge of the genomic complexity have been filled by the identification of several families of long RNAs [3, 4]. In particular, massive parallel sequencing technology has revealed that noncoding regions of the human genome are also dysregulated in various types of cancer [5]. Statistics from genome sequencing projects suggest that up to 80% of the human genome is dynamically and differentially transcribed into functional RNAs, whereas less than 2% of the entire genome contains genes that code for proteins [6]. This results in the generation of a rich repertoire of non-coding transcripts. Non-coding RNAs, which are RNA transcripts that do not code for proteins, can be broadly classified into two categories based on length, small ncRNAs (< 200 nucleotides) and long ncRNAs (> 200 nucleotides). The major classes of small ncRNAs regroup into microRNAs (miRNAs), small nuclear RNAs (snRNAs), piwi-interacting RNAs (piRNAs), and small nucleolar RNA (snoRNAs) [7, 8]. The lncRNA group comprises both linear lncRNAs (named by default as lncRNAs) and circular RNAs (circRNAs) (Fig. 1). Small ncRNAs have been fascinating the scientific community for the last couple of decades. In particular, miRNAs which fine-tune the key biological processes via regulating messenger RNA (mRNA) translation, are now recognized as powerful and ubiquitarians regulators of gene expression [9]. Nevertheless, miRNAs are just a small facet of noncoding RNAs scratching only the surface of the RNA world.

**Fig. 2:**
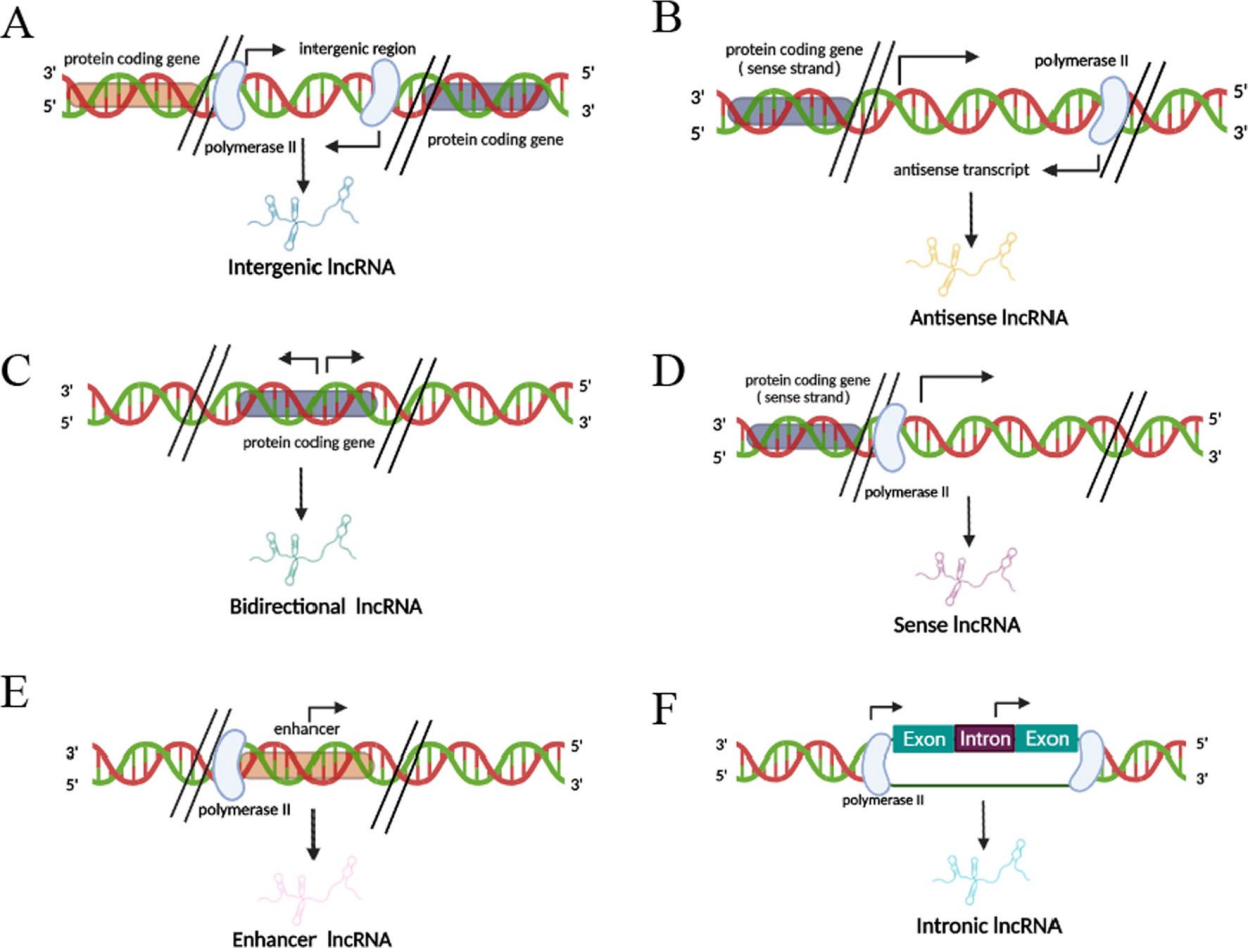
Classification according to lncRNA location with respect to protein-coding gene. **A** Intergenic lncRNA, transcribed from the region between two protein-coding genes. **B** Antisense lncRNA, transcribed from the antisense strand of protein-coding genes. **C** Bidirectional lncRNA, transcribed from the region between two protein-coding genes. **D** Sense lncRNA, transcribed from the sense strand of protein-coding genes. **E** Enhancer lncRNAs, transcribed from enhancer regions of protein-coding genes. **F** Intronic lncRNA, transcribed entirely from introns of protein-coding genes

**Fig. 3:**
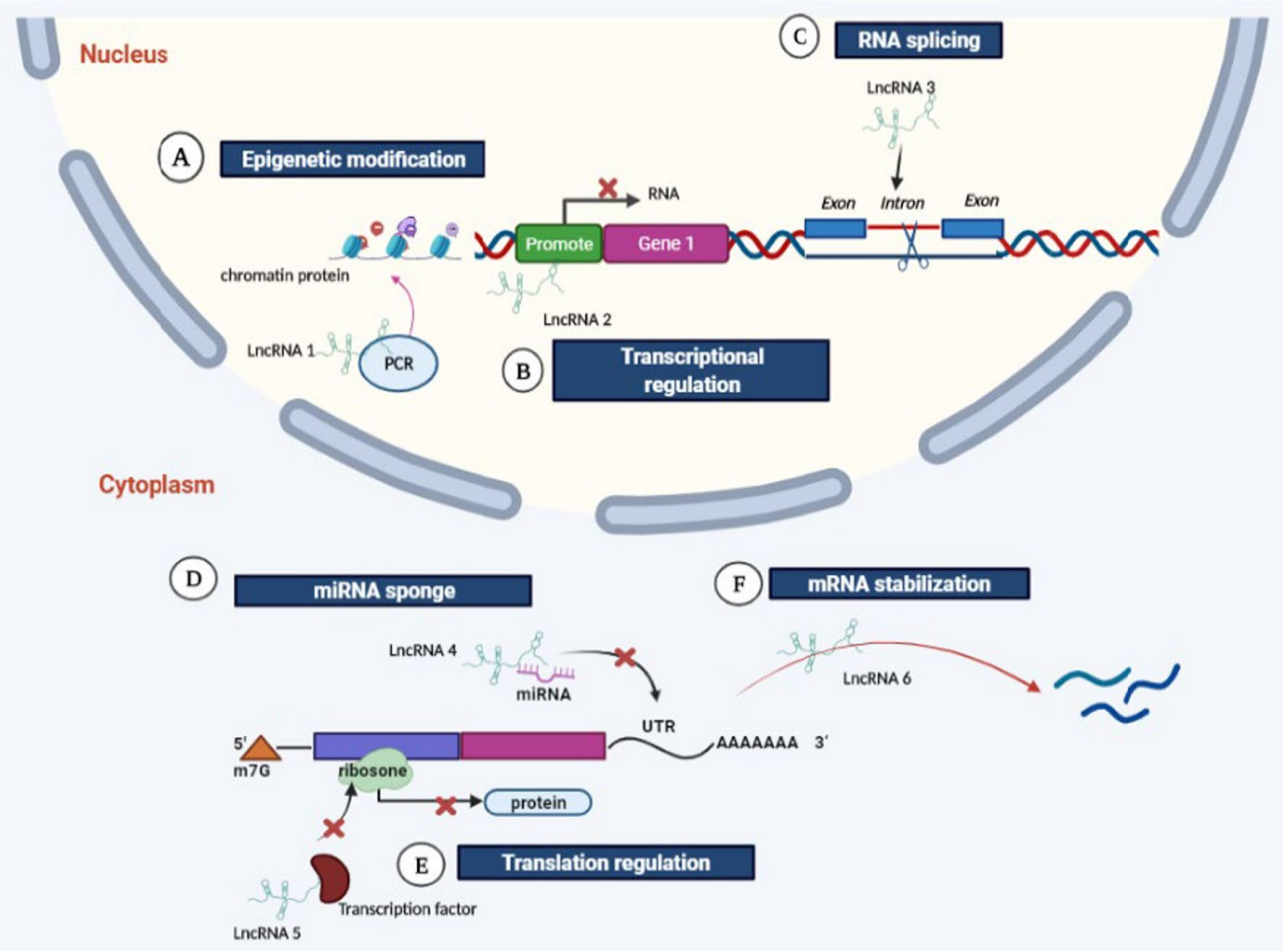
General functions and mechanisms of lncRNAs. **A**–**C** lncRNA’s function in the nucleus, **D**–**H** lncRNA’s function in the cytoplasm. **A** LnRNA1 functions to modify chromatin protein through recruiting the complex of PRC2, resulting in suppression of gene expression. **B** LnRNA2 binds to the promoter of gene A and blocks the binding of transcription factors, thus affecting transcription initiation of gene A. **C** LnRNA3 functions to modulate the pool of modified splicing factor, thereby influencing gene splicing. **D** LnRNA4 acts as miRNA sponge and interacts with miRNA to form a complex and, thus, inhibits the binding of miRNA to the 3' UTR of target mRNA. **E** LnRNA5 interacts with translational factors to inhibit translation. **F** LnRNA6 serves as natural antisense inhibitor to promote degradation of mRNA

Long ncRNAs, once depicted as ‘the dark matter of the genome’, is gaining more and more interest among the scientific community. Similar to small ncRNAs, most lncRNAs are transcribed by RNA polymerase II, 5′-capped, polyadenylated at their 3′ ends and spliced. Nevertheless, lncRNAs can fold into specific secondary and tertiary structures which enable them to have both RNA and protein-like functions [10, 11]. From a genetic point of view long, they can be classified as bidirectional, intronic, enhancer, antisense, sense, and intergenic based on the relationship with protein-coding genes [12] (Fig. 2). Depending on the subcellular localization, lncRNAs have profound impacts on transcriptional and post-transcriptional control. Nuclear lncRNAs can function as epigenetic modulator, transcription regulator and RNA processor while cytoplasmic lncRNAs act as post-transcriptional regulators to affect mRNA stability and translation [13] (Fig. 3). It is not surprising, then, that differentially regulated lncRNAs implicated in several human disease, their functions in malignant transformation is of particular interest [14]. A growing number of studies revealed the dysregulation of various lncRNAs in tumor samples compared with their normal counterparts hallmarked major cancer properties, such as sustaining proliferative signaling, evading growth suppression, induction of angiogenesis, activating invasion and metastasis, resisting cell death and enabling replicative immortality [15–18]. These findings, and especially the high tissue specificity, sensitivity and reliable stability, established the rationale for assessing lncRNAs as possible biomarkers and therapeutic targets. Further, the lack of translation, rapid turnover and low expression levels may facilitate faster effects at lower doses [19]. In general, lncRNAs could serve as oncogenes or tumor suppressor genes by transcriptional regulation, posttranscriptional mediation, and other regulatory functions demonstrated by a distinctive cross-talk between lncRNAs and miRNAs to exert their biological functions.

High expression in hepatocellular carcinoma, also known as hepatocellular carcinoma upregulated EZH2-associated lncRNA (HEIH), is a recently identified intergenic lncRNA mapped on chromosome 5q35.3 (https://www.ncbi.nlm.nih.gov/)*.* The gene sequence of HEIH is more than 1000 nucleotides in length, and can be transcribed to form transcript NR_045680.1 (http://www.noncode.org/). Although HEIH RNA molecules can be detected in both the cytoplasm and nuclease, HEIH primarily exists in cytoplasm [20]. HEIH was firstly characterized and investigated in HBV-related hepatocellular carcinoma as an oncogenic lncRNA resulting from its inhibition for cell differentiation in G0/G1. It was postulated that the HEIH was associated with enhancer of zeste homolog 2 (EZH2), and this association was required for the repression of the EZH2 target genes, involving p15, p16, p21 and p57 [21]. Subsequently, accumulating studies have reported that HEIH is abnormally expressed in a variety of tumors and its dysregulation is closely correlated with carcinogenesis, affecting the prognosis of cancer patients. Taken together, the above findings suggest that HEIH is a functional lncRNA in tumor progression. What’s more, multiple studies have indicated that HEIH plays a key role in different cancers through a variety of complex mechanisms. The present review briefly summarized the current researches of clinical significance and underlying mechanism of HEIH in the tumorigenesis and progression of human cancers.

## HEIH in various human cancers

### Digestive system neoplasms

**Fig. 4:**
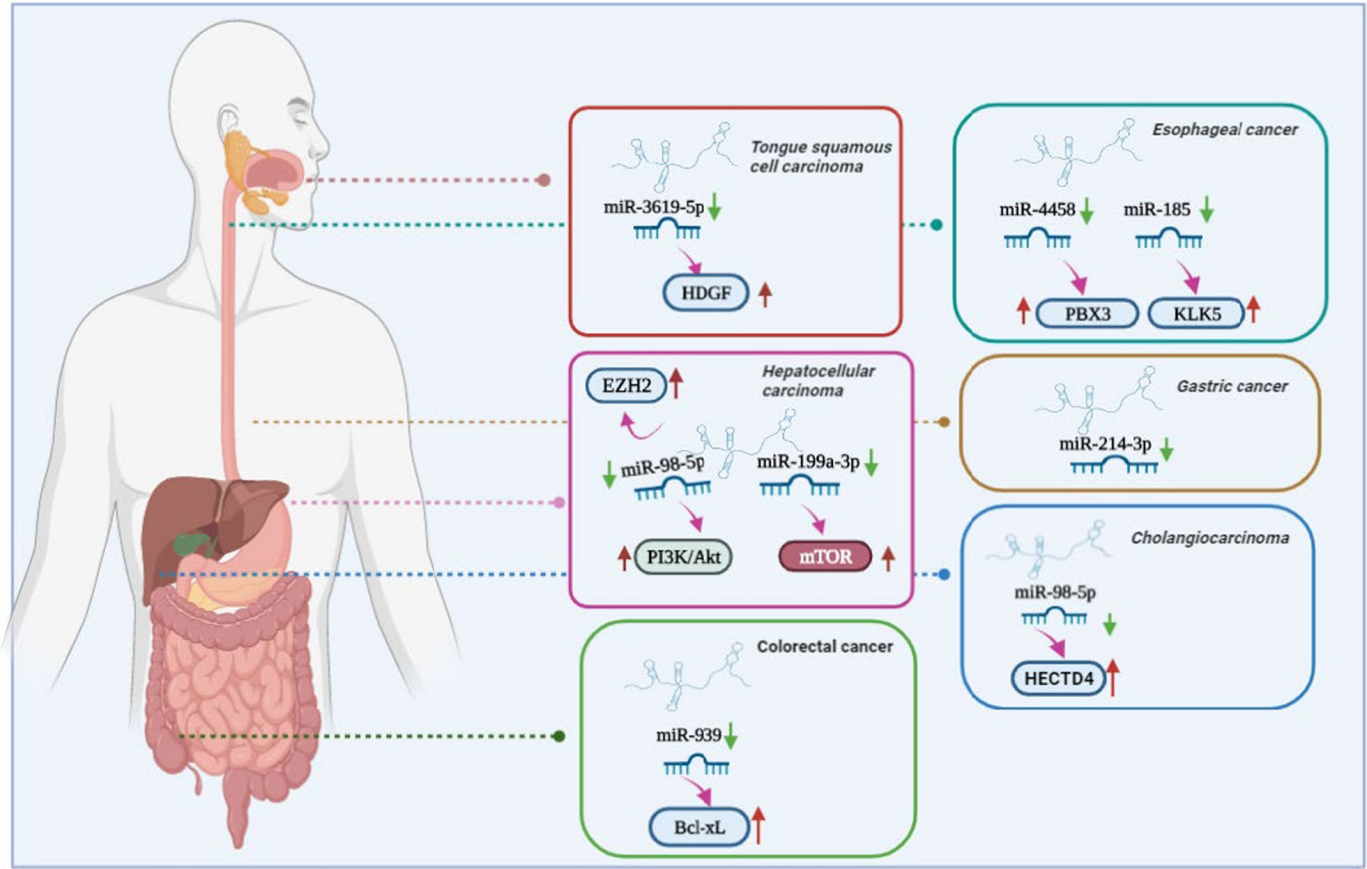
Mechanism of HEIH in regulating digestive system cancers

Tumors of the digestive system, when combined together, account for more new cases and deaths per year than tumors arising in any other system of the body and their incidence continues to increase [22]. Mounting evidences have confirmed that HEIH as an oncogene, is abnormally expressed in numerous digestive system cancers, such as hepatocellular carcinoma (HCC) [21, 23–27], tongue squamous cell carcinoma(TSCC) [28], gastric cancer (GC) [29–31], esophageal cancer (EC) [32–34], colorectal cancer (CRC) [35], and cholangiocarcinoma [20]. But so far, no studies have yet examined the function of HEIH in cancers of the pancreas and other digestive system organ. In all studied cancers mentioned above, the results indicated that HEIH was overexpressed in the tumor tissues compared with adjacent normal tissues, and the dysregulated expression was correlated with aggressive clinicopathological features and unfavorable prognosis. In these cases, HEIH was involved in various cellular function including cell proliferation, apoptosis, migration, metastasis, and invasion. Besides, the cellular mechanisms by which HEIH mediates oncogenic activity was complicated and associated with multiple factors. In particular, effects of HEIH on HCC cell proliferation was controversial. Yang et al. initially demonstrated that HEIH has capacities in regulating HCC cells proliferation to facilitate tumor growth through enhancer of EZH2 [21]. Consistence with previous study, a later study demonstrated that HEIH functioned to HCC cells growth and metastasis which might be partially through negative regulation of miR-199a-3p, and thereby positively activated mTOR signaling [23, 25]. However, another research reported that aberrant expression of HEIH was conducive to invasion of HCC cell lines, but could not impact the proliferation of HCC cells [27]. Of note, upregulation of HEIH also acted as a sponge for miR-98-5p, leading to activation of the PI3K/AKT signaling pathway, which conferred an advantage to sorafenib resistance in HCC [24]. A similar result has proposed the high expression of HEIH in TSCC, and HEIH overexpression upregulated HDGF expression by directly targeting miR-3619-5p, thus leading to enhancement of cisplatin resistance, which promoted cell proliferation and inhibited apoptosis [28]. In GC, HEIH was highly expressed in gastric cancer samples and closely related to the TNM stage, suggesting HEIH could serve as a marker for early diagnosis of GC malignancy. In addition, a bioinformatics analysis found that miR-214-3p might be a target of HEIH and silencing of HEIH could suppress gastric cancer cell proliferation, migration and invasion [30]. Wang et al. examined EC and their results showed that HEIH was first found to be notably elevated in EC tissues and cells, and depleting HEIH depressed the viability and invasion of EC cells by sponging miR-4458 and upregulating PBX3 [33]. Other researchers proffered that HEIH had higher expression in EC cancer samples than normal samples, and tumor size, infiltration depth, and TNM staging were correlated with HEIH expression that are indicative of more advanced disease and poor prognosis. The increased HEIH expression promoted EC cell proliferation, invasion, and migration, inhibited cell apoptosis rate in vitro, as well as promoted tumor growth tumor volume and weight in vivo [32, 33]. More precisely, HEIH functioned as a sponge that bound with miR-185 to regulate KLK5 expression, or HEIH directly suppressed the expression of p53 through EZH2, thus promoting tumor progression [34]. Studies of CRC found that HEIH was significantly overexpressed in CRC tissue specimens and cell lines, and increased HEIH was positively associated with greater tumor size, invasion depth, and poor prognosis. In addition, HEIH overexpression promotes cell proliferation and inhibits apoptosis in vitro, and promotes CRC tumor growth in vivo via counteracting miR-939-mediated transcriptional repression of Bcl-xL [35]. Studies of cholangiocarcinoma reported that increased expression of HEIH promoted tumorigenesis and progression by modulating miR-98-5p/HECTD4 axis [20]. Figure 4 summarizes the regulatory mechanisms of HEIH in digestive system cancers. Further studies are needed before HEIH can be used as a reliable serum biomarker and therapeutic target for these cancers.

### Reproductive system cancers

Endometrial cancer and ovarian cancer (OC) are some of the most commonly diagnosed neoplasms in gynecological malignancies and female with the two gynecological cancers have especially poor prognoses, furthermore with five-year survival rates below 45% [36]. Prostate cancer is the second most frequently diagnosed malignance in males worldwide, the incidence and mortality continues to rise and pose a major health problem [37]. Numerous lncRNAs showed evidence of function in oncogenic transformation or tumor progression of reproductive system cancers including endometrial, OC, and prostate cancer [38, 39]. Chemotherapy paclitaxel is the main front-line chemotherapy in endometrial cancer based mainly on its favorable toxicity profile and superior response rate. However, the therapeutic efficacy was limited resulting from acquired resistance [40]. Guo et al. showed HEIH, the dysregulation of which involved in production of drug-resistance, was significantly upregulated in paclitaxel-resistant endometrial cells. The increased HEIH promoted chemo-resistance of endometrial cancer cells and enhance cell proliferation and viability, whereas silencing HEIH successfully improved sensitivity to paclitaxel and repressed cell physiological process [41].

Epithelial ovarian cancer (EOC) accounts for approximately 90% of all OC cases and a major cause of death from gynecologic cancers. The majority of EOC is typically diagnosed at an advanced stage due to rapid progression of the disease and nonspecific clinical symptoms [42]. Study of EOC identified HEIH that having the maximum number of connections with related mRNAs upon the lncRNA-mRNA co-expression network analysis, suggesting HEIH play crucial roles in regulating gene expression and protein translation in EOC. Further research showed there was a significant difference of HEIH expression between normal ovary cell lines and OC cell lines. Functionally, HEIH silencing significantly reduced the viability and proliferative activity of OC cells, and wound-healing and transwell migration assays indicated key roles for HEIH in promoting OC cell invasion and migration [43]. Another study confirmed that HEIH expression was up-regulated in OC tissues and cell lines. Besides, elevated HEIH expression was related to poor prognosis. Mechanically, HEIH accelerated cell proliferation, migration and invasion, whereas inhibited cell senescence via targeting the miR-3619-5p/CTTNBP2 axis [44]. It is tempting to conclude that bias towards up-regulation of HEIH in cancer may results from the above large-scale practice discovering HEIH from various cancer samples. Bawa et al. performed an integrative analysis of lncRNAs across multiple RNA-seq datasets pertaining to cancer from public repository to address this bias. Interestingly, they observed an isoform of HEIH which is reduced expression in prostate cancer tissues compared with normal prostate tissues, and the abnormal expression may be functionally relevant [45]. From what have been discussed, HEIH maybe a valuable predictor and a potential target for treatment of reproductive system cancers. However, the biological functions and molecular mechanisms of HEIH are not yet fully understood. Further well-designed studies with large sample sizes are needed to understand the role of HEIH and elucidate the molecular mechanisms in reproductive system cancers.

### Breast cancer

**Fig. 5:**
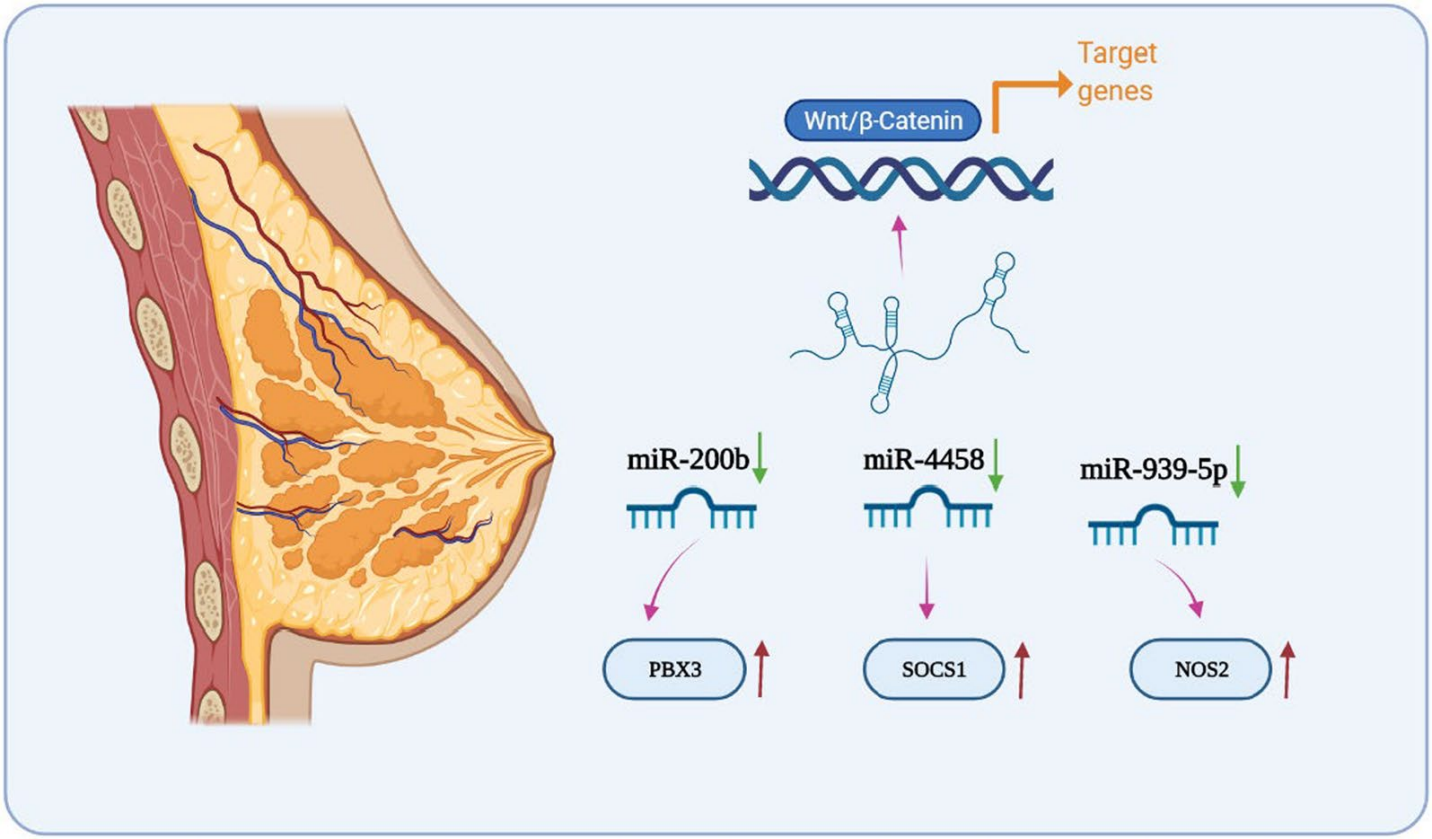
Mechanism of HEIH in regulating breast cancer

According to the American Cancer Society, breast cancer is the most frequently diagnosed women's malignant tumor in 2020, accounting for about 30% of female cancers [46]. Hence, screening out reliable potential biomarkers and therapeutic targets for breast cancer should be a priority. Alterations of certain lncRNAs in breast cancer activate malignancy pathways and induce cancer cell proliferation and progression, which increasingly attracted widespread attention [47]. Recent studies have investigated the potential function and molecular mechanisms of HEIH in breast cancer. High expression levels of HEIH were detected in the breast cancer tissues and cell lines, and the increased HEIH expression had significant correlations with adverse pathology and poor clinical outcome. More specifically, HEIH was positively associated with the tumor size, tissue invasion, malignancy status and poor prognosis, suggesting HEIH functioned as an oncogene in breast cancer [48]. Wang et al. examined the mechanism by which HEIH contribute to tumorigenesis and metastasis by regulating microRNA-200b, enhanced the expression of PBX3, and subsequently activated the Wnt/β-catenin pathway [49]. Triple-negative breast cancer (TNBC) is characterized by lack of progesterone receptors, estrogen receptors and human epidermal growth factor receptor-2, and it has the worst clinical outcome among all types of breast cancer due to high metastatic risk, extensive heterogeneity, and the absence effective molecular target [50]. Li et al. validated that HEIH acted as an oncogene that was overexpressed in TNBC patients and cell lines, and the higher level of HEIH expression was relevant with advanced clinical stage. Mechanistically, experiments performed in vitro demonstrated HEIH promoted TNBC cell proliferation and inhibited apoptosis through regulating miR-4458/SOCS1 axis [51]. Other investigations found that HEIH was an upstream suppressor of miR-939-5p, which subsequently elevated NOS2 and NO production to drive TNBC progression [52]. Taken together, all above findings categorized HEIH as a potential oncogenic lncRNA in breast cancer, which may be used as a novel diagnostic and prognostic indicator for this disease (Fig. 5).

### Non-small cell lung carcinoma

Lung cancer remained the most frequently identified tumor and introduced as the major cause of the cancer-associated mortality [53]. Non-small cell lung cancer (NSCLC), which represented approximately 85% of all lung cancer cases, was regarded as the primary subtype of lung carcinoma. Despite recent progress in targeted molecular therapies, more than 40% patients with NSCLC still presented with late stage at the time of diagnosis, with a low 5-year survival rate [54]. For this reason, specific diagnostic biomarkers and molecular targets could be very helpful in the early detection and prognosis. Recent studies demonstrated that lncRNAs have fundamental roles in the biochemical and cellular processes that underlie the development of NSCLC [55]. Jia et al. identified HEIH was notably upregulated in NSCLC tissues and cell lines compared with compared with the normal ones. Moreover, HEIH was strongly associated with the progression of cell proliferation, migration and invasion, thus leading to increased tumorigenesis and poor prognosis, suggesting that HEIH may have potential as a novel therapeutic strategy in NSCLC [56].

### Other cancers

HEIH is also involved in the pathogenesis of retinoblastoma [57], melanoma [58], nasopharyngeal carcinoma (NPC) [59] and head and neck squamous cell carcinoma (HNSCC) [60]. HEIH has been identified as an oncogene in retinoblastoma where up-regulated HEIH was associated with TNM stage, optic nerve invasion and choroidal invasion, predicting an unfavorable overall survival. Loss-of function assays showed that HEIH knockdown significantly suppressed retinoblastoma cell proliferation, migration and invasion [57]. Similarly, HEIH have been shown to be induced in melanoma tissues and cell lines, and positively correlated with advanced clinical stages and poor outcome. Mechanistically, HEIH promoted melanoma cell proliferation, migration and invasion by directly inhibiting miR-200b/a/429 transcription [58]. HAN et al. reported that HEIH was high expressed in both NPC tissues and cells. Forced up-regulation of HEIH facilitated cell proliferation, migration and invasion by sponging miR-193a-5p to modulate CDK8 expression [59]. Surprisingly, Haque et al. found that HEIH expression was decreased in HNSCC tissues compared normal tissues through RNA-sequencing data and qRT-PCR, while the underlying function and mechanism of HEIH in HNSCC was unclear, should be further investigated in the future [60]. Although the studies for these other cancers were not fully explored to the maximum extent and the data are more limited, our review emphasized that the abnormal expression was closely interrelated with tumor progression, indicating HEIH was extraordinarily promising as a viable drug target.

## Mechanistic model of HEIH in human cancer

Generally, LncRNAs exist both in the nucleus and cytoplasm, and the versatile biological functions of LncRNA are closely associated with their subcellular localization [61]. In the nucleus, lncRNAs can function as either transcriptional or epigenetic regulators of the genome by affecting transcription factors’ function and guiding chromatin modifiers, respectively. However, in the cytoplasm, lncRNAs regulate gene expression through allosteric regulation, acting as miRNAs sponges, controlling cell signaling, and also modulate posttranscriptional events. Majority of lncRNAs are located in nucleus, in part owing to inefficient splicing and polyadenylation and susceptibility to degradation by exosomes on chromatin, which is consistent with the major functions of lncRNA for epigenetic regulation of gene expression [62]. Interestingly, although HEIH can be detected in both the cytoplasm and nucleus, HEIH was localized predominantly in the cytoplasm, thus HEIH-mediated gene expression may mainly take place at post-transcriptional levels.

### Function of HEIH as a ceRNA

Competing endogenous RNAs (ceRNAs) are RNA transcripts that act as miRNA sponges competing for shared miRNAs through miRNA response elements (MREs). The ceRNA regulatory network provides a main mechanism by which HEIH can post-transcriptionally regulate gene expression through abolishing miRNA endogenous suppressive effects on key targets [63]. Several articles have demonstrated that HEIH sequester miRNAs through MREs located in its sequence, thus relieving the inhibitory effects of tumor suppressor miRNAs on oncogenic targets, and exerting its oncogenic function on various cancers. Coherently with this function, HEIH was found to promote malignant phenotypes by competitively binding miR-4458 to upregulate the expression of SOCS1and PBX3 in breast cancer and esophageal cancer, respectively [33, 51]. Similarly, HEIH triggered ovarian cancer and tongue squamous cell carcinoma development by sponging miR-3619-5p, and consequently upregulating the miR-3619-5p targets CTTNBP2 and HDGF [28, 44]. Nafea et al. and Cui et al. showed HEIH could directly interact with miR-939 to counteract the effects of miR-939 on the transcription of Bcl-xl in colorectal cancer [64] and the production of NO_2_ in TNBC [52]. Wan et al. verified the ceRNA network among HEIH, miR-98-5p and HECTD4 to promote cholangiocarcinoma tumorigenesis [20]. Additionally, HEIH was proved to be a ceRNA of miR-193a-5p to modulate CDK8 expression in NPC [59]. And HEIH could promote WEE1 expression by binding and sequestering its major negative regulator miR-194-5p in retinoblastoma cells [57]. Finally, Jiang et al. and Wu et al. proved that HEIH could directly interact with miR-214-3p and miR-199a-3p in GC and HCC, respectively [25, 30].

### P53-dependent regulation

The p53 gene is the single most frequently altered gene involved in cancer formation and progression. Functionally, p53 acts mainly as a tumor suppressor via modulating a variety of transcriptional and non-transcriptional activities that lead to the tight control of cell proliferation, apoptosis, senescence, and DNA repair [65]. Generally, regulation of p53 level takes place primarily at translation, and post-translational modifications and protein stability [66]. To date, the available evidence of transcriptional regulation of p53 by lncRNAs was scarce, probably as the p53 mRNA level often remained consistent in the cell [67]. Of great interest, Ding et al. demonstrated that HEIH was involved in transcriptional repression through recruitment of PRC2 complex protein EZH2 to p53 promoter, which proposed a model that HEIH could associate with chromatin-modifying complexes to regulate p53 expression in EC [34]. Furthermore, Ma et al. demonstrated that silence of HEIH contributed to cell viability arrest and induction of apoptosis by upregulating the expression of p53 in HCC [25].

### HEIH is involved in several signaling pathways

The interwoven signaling pathways present in many human cancer types complicate the development of targeted therapies or chemotherapeutic agents [68]. Although still largely unexplored, it has been suggested that HEIH was essential mediator of intracellular signaling pathways in several tumors. Zhao et al. found that the expression Wnt signaling related proteins wnt1, c-Myc, cyclin-D1, and β-catenin was remarkably reversed after suppression of HEIH, suggesting HIEH could induced the activation of Wnt/β-catenin pathway to promote breast cancer development [49]. Moreover, aberrant activation of mTOR signaling has been implicated in HCC, and HEIH silence exhibited inhibitory effects on the activation of mTOR signaling, leading to decelerate tumor growth and increase survival [25]. HEIH also implicated in regulation of AKT activity through regulating miR-98-5p in sorafenib-resistant HCC cells. The significance of AKT in cancer is further supported by the findings that NF-κB served an important AKT effector. Thus, it is conceivable that AKT associated HEIH may also affect NF-κB activity to certain degree, further highlighting the importance of these AKT associated HEIH [24]. As an important feature of lncRNAs in serving as a scaffold, lncRNAs can interact with different kinases in cytoplasm that are often critical to cell signaling [69]. Coherently with this feature, HEIH was proved to mediated Mitogen-activated protein kinase (MAPK) signaling pathway in restoring chemo-sensitivity of endometrial cancer. Future studies on the regulatory and biological roles of HEIH in cancer signaling will define the future of lncRNA-based clinical applications [41].

## The clinical value of HEIH in various cancers

Generally, metastases occur only at an advanced stage of cancer, and if diagnosed at earlier stages could lead to better outcomes for most cancer. The frequently used cancer diagnostic markers and tools include endoscopic ultrasound, non-invasive imaging, and cytology based on fine-needle aspiration. These methods are useful for rendering an accurate diagnosis in many cases, however each with unique disadvantages. Ultrasonography has poor sensitivity for detection of smaller tumors, and widespread use of fine-needle aspiration cytology and non-invasive imaging were limited because they come with invasive, high cost and associated risks of radiation exposure. Therefore, non-invasive or minimally invasive methods with the potential for routine and inexpensive screening are required [70]. LncRNA can be easily obtained and analyzed from body fluids such as serum and urine, which demonstrate the superiority of LncRNA-based diagnostic strategies [14]. Currently, lncRNA CCAT1 (ClinicalTrials.gov Identifier: NCT04269746) and HOTAIR (ClinicalTrials.gov Identifier: NCT03469544) are in clinical trials for colorectal and thyroid cancer diagnostic biomarkers studies [71]. Recent studies have suggested that HEIH was stable enough to be detected in the serum of cancer patients [26]. Additionally, the fact that HEIH was highly dysregulated in several cancer types, emerging as a novel marker in cancer diagnosis.

Prognosis is a forecasting of the probable course and outcome of a disease, especially of the chances of recovery or survival from the disease. Factors such as tumor type, its location in the body, tumor size, tumor grade and cancer relapse affect prognosis [72]. The increased expression of HEIH promoted tumor development and is strongly correlated with clinicopathological parameters, including lymph node metastasis, as well as tumor size, differentiation, clinical stage and recurrence, and thus could be used as a prognostic marker for several cancers listed in Table 1. Notably, Kaplan–Meier analysis indicated a negative correlation between HEIH expression levels and overall survival rates in patients with breast cancer, OC and retinoblastoma [44, 48, 57]. Jointly, the above findings indicate that HEIH might represent an independent parameter for predicting prognosis in various cancers, although further large trials are still required for confirmation.

**Table 1:** Functional characterizations of HEIH in multiple human cancers

Cancer types	Expression	Role	Targets/regulators and related pathways	Function	Clinical correlation	Reference (PMID)
Hepatocellular carcinoma	Upregulated	Oncogenic	miR-98-5p/PI3K/AKT, miR-199a-3p/mTOR, EZH2	Proliferation, differentiation, Migration, apoptosis, metastasis, cell cycle	Prognosis, recurrence, drug resistance	29286922 32572917 32821157 21769904
Tongue squamous cell carcinoma	Upregulated	Oncogenic	miR-3619-5p/HDGF	Proliferation, apoptosis	Drug-resistance	33130420
Gastric cancer	Upregulated	Oncogenic	miR-214-3p	Proliferation, migration, invasion, apoptosis	Prognosis, TNM stage, diagnosis, tumor size, overall survival time	33015781 33226411 33877891
Esophageal cancer	Upregulated	Oncogenic	miR-185/KLK5, miR-4458/PBX3, P53	Proliferation, invasion, migration, apoptosis	Prognosis, tumor size, infiltration depth, TNM stage, overall survival time, drug-resistance	33223519 32449803 32729661
Colorectal cancer	Upregulated	Oncogenic	miR-939/Bcl-xL	Proliferation, apoptosis	Tumor size, invasion depth, poor prognosis	29081216
Cholangiocarcinoma	Upregulated	Oncogenic	miR-98-5p/HECTD4	Proliferation, migration, invasion	Tumor size	32062383
Endometrial cancer	Upregulated	Oncogenic	MAPK	Proliferation, viability, apoptosis	Drug-resistance	31650338
Ovarian cancer	Upregulated	Oncogenic	miR-3619-5p/CTTNBP2	Proliferation, migration, invasion, senescence	Prognosis	33110047
Prostate cancer	Downregulated	–	–	–	–	25933431
Breast cancer	Upregulated	Oncogenic	miR-200b/PBX3, Wnt/β-catenin, miR-4458/SOCS1, miR-939-5p/NOS2	Proliferation, apoptosis, metastasis, viability, migration and invasion	Prognosis, TNM stage, tumor size, lymph node metastasis, overall survival time	31526439 31456112 33368266
Non–small cell lung carcinoma	Upregulated	Oncogenic	–	Proliferation, apoptosis, migration, invasion	–	30230600
Retinoblastoma	Upregulated	Oncogenic	miR-194-5p/WEE1	Proliferation, migration, invasion	TNM stage, optic nerve invasion, choroidal invasion, overall survival time	33262604
Melanoma	Upregulated	Oncogenic	miR-200b	Proliferation, migration, invasion	Prognosis, clinical stages	28487474
Nasopharyngeal carcinoma	Upregulated	Oncogenic	miR-193a-5p/ CDK8	Proliferation, migration, invasion	–	33577031
Head and neck squamous cell carcinoma	Downregulated	–	–	–	–	29575229

Apart from emphasizing the implications of HEIH in cancer diagnosis and prognosis, HEIH can also be considered for improving therapeutic efficacy. The development of chemoresistance is the main cause of treatment failure. However, therapeutic resistance could be reversed by improving the therapeutic sensitivity of tumors by modulating a critical cell signaling pathway that confers resistance. Paclitaxel-mediated upregulation of HEIH in endometrial cancer cells as a possible mechanism for resistance, and HEIH silence restored chemo-sensitivity to paclitaxel by suppressing MAPK signaling pathway [41]. Similarly, HEIH conferred an advantage to sorafenib resistance in HCC by the activation of PI3K/AKT pathway [24]. Recent reports have suggested that exosomes, nanosized (30–100 nm) membrane micro-vesicles, can act as messengers in the interstitial to establish communication between cancer cells and basal cells [73]. Exosomal HEIH derived from drug-resistant cells could be transmitted into sensitive cells and made sensitive cells more resistant to drug treatment [28]. Thus, HEIH held strong promise towards the discovery of novel diagnostics and therapeutics for various cancer.

## Conclusions and future perspectives

Over the past decade, nearly 8000 lineage and/or cancer-specific lncRNAs have been nominated, which open up a whole new range of possibilities for the diagnostics and treatment of cancer [74]*.* For instance, PCA3 (prostate cancer gene 3) is prostate-specific, prognostic biomarker which is detectable in urine sediments with levels that correspond to the severity of prostate cancer [75]. In addition, PRNCR1 (prostate cancer noncoding RNA 1) and PCGEM1 (prostate cancer gene expression marker 1) are exclusively associated with prostate cancer and are potential diagnostic markers [76]. The same is true for HULC (highly upregulated in liver cancer) whose expression is highly expressed in primary HCC and hepatic metastases of CRC, but is not found in primary colon cancers or in non-liver metastases [77]. Similarly, MALAT1 (metastasis associated in lung adenocarcinoma transcript 1) can serves as an independent prognostic parameter for patient survival in early-stage lung cancers [78]. These few examples of lncRNAs show already the great value of these newly discovered transcripts. Moreover, novel lncRNAs such as HIEH are still being discovered, which add new regulatory layers to the tumor pathophysiology. With these characteristics, lncRNAs hold the promise of tailored therapeutic applications either by inhibition or restoration of lncRNAs, and an interesting future frontier will be the pursuit of anti-tumor strategies that focus on RNA as target molecule.

Advances in the human transcriptome have improved our comprehensive understanding of gene regulation in cancer. RNA-based therapeutic approaches to treat cancer are becoming increasingly utilized in the clinics and demonstrated durable clinical benefit in several tumor types. In Phase I-II clinical trial of pancreas, ovarian and bladder cancer patients, BC-819, a double stranded DNA vector carrying the promoter of lncRNA H19 and coding sequence of diphtheria toxin showed highly promising results, suggesting the possibility of lncRNA as a new therapeutic tool [79, 80]. At present, the research on HEIH is still at its infancy that eventually created many research opportunities to fully explore this lncRNA. Without detailed understanding on the structure and functions of HEIH, developing HEIH-based therapies is like "shooting in the dark". However, huge challenges remained, notably the lacking of proper animal models for testing and validations prior to clinical trials, particularly for confronting HEIH tend to be poorly conserved across species. Given the low sequence conservation, functional characterization of primate-specific HEIH in non-primate models, such as mice, can yield ambiguous results, which restrict the scope of research. Thus, there is a daunting task to identify homologs in other species [62, 81]. Nevertheless, the prospects and clinical significance of HEIH in human cancers cannot be disparaged in the long run. A distinctive feature of HEIH is its high cell-type specificity, making it possible for it to selectively kill tumor cells without damaging normal cells. In addition, abnormally expressed HEIH can be extracted in a noninvasive manner, which high lightings its potential to be more economical and less harmful. Compared to protein-based anti-tumor drugs, lncRNA are more refined and less toxic, and the low expression of lncRNA means that only a small number of inhibitors are needed to make a difference. Besides, bioinformatics and computational tools provide new opportunities for lncRNA biomarker development [82]. In summary, HEIH bring a new paradigm in cancer research, and may emerge as a star in diagnosis and therapy in the future.

## Data Availability

All data are included in the article.

## Abbreviations

LncRNA: Long noncoding RNA
SnRNAs: Small nuclear RNAs
SnoRNA: Small nucleolar RNA
SiRNA: Small interfering RNA
PiRNA: Piwi-interacting RNA
CircRNAs: Circular RNAs
MiRNA: Micro RNA
EZH2: Enhancer of zeste homolog2
HEIH: Hepatocellular carcinoma upregulated EZH2-associated lncRNA
HCC: Hepatocellular carcinoma
TSCC: Tongue squamous cell carcinoma
GC: Gastric cancer
EC: Esophageal cancer
CRC: Colorectal cancer
OC: Ovarian cancer
TNBC: Triple-negative breast cancer
NSCLC: Non-small cell lung cancer
NPC: Nasopharyngeal carcinoma
HNSCC: Head and neck squamous cell carcinoma
CeRNAs: Competing endogenous RNAs
PCA3: Prostate cancer gene 3
PRNCR1: Prostate cancer noncoding RNA 1
HULC: Highly upregulated in liver cancer
MALAT1: Metastasis associated in lung adenocarcinoma transcript 1
PCGEM1: Prostate cancer gene expression marker 1
